# Agreement between heart rate variability‐derived and lactate/ventilatory thresholds during a 4‐min stepwise incremental cycling test in male adults

**DOI:** 10.14814/phy2.70777

**Published:** 2026-02-16

**Authors:** Olieslagers Anton, Müller‐Jabusch Yoram, Vancoillie Margot, Delen Emma, de Beukelaar Toon

**Affiliations:** ^1^ Faculty of Movement and Rehabilitation Sciences KU Leuven Leuven Belgium; ^2^ KU Leuven Institute of Sports Science Leuven Belgium

**Keywords:** endurance training, HRV threshold, incremental test, lactate threshold, threshold determination, ventilatory threshold

## Abstract

Accurate exercise intensity thresholds are key for endurance training prescription. The non‐linear heart rate (HR) variability (HRV) index DFAa1 has been proposed as a threshold determination marker, with DFAa1 values of 0.75 (HRVT1) and 0.5 (HRVT2) corresponding to aerobic and anaerobic thresholds, respectively. This study investigated the agreement between these novel HRVT's and the gold standard blood‐lactate (LT) and ventilatory thresholds (VT). Twenty‐one trained participants completed a 4‐min stepwise cycling test (starting 40 W, +30 W/stage) with continuous HR and gas exchange measurement. Capillary blood lactate was sampled after each stage. Agreement between corresponding thresholds was assessed for HR, power output (PO), and oxygen uptake (V̇O_2_) using (intraclass) correlation coefficients (ICC) and ANOVA. HRVT1 demonstrated poor agreement with LT1/VT1, while a more personalized determination method (HRVT1_pers_) rendered trivial to moderate correlations and poor ICC. Both exhibited substantial mean bias relative to LT1/VT1. Conversely, HRVT2 demonstrated stronger agreement with LT2/VT2, particularly for PO and V̇O_2_, with smaller mean biases but wide limits of agreement. ANOVA revealed significant HR differences between VT2 and LT2 and between VT2 and HRVT2. These findings support HRVT2 validity as a surrogate for LT2/VT2 in 4‐min stepwise protocols, whereas HRVT1 demonstrates insufficient reliability for determining LT1/VT1.

## 1 INTRODUCTION

Accurate threshold determination and load monitoring are essential for optimizing endurance training and performance. To prescribe exercise intensity accurately, training zones are typically determined using incremental exercise tests in both research and applied settings (Bentley et al., 2007; Gronwald et al., 2020; Seiler & Kjerland, 2006). Incremental tests incorporating blood lactate and/or gas exchange measurements are considered the gold standard for determining exercise thresholds (Jamnick et al., 2020). From these measurements, the transition from moderate to heavy exercise intensity is commonly designated as the aerobic threshold (AeT), represented by the first lactate (LT1) or first ventilatory threshold (VT1). Exercise below AeT is characterized by stable, near‐baseline blood lactate and oxygen uptake (V̇O_2_) levels (Jamnick et al., 2020). Conversely, the anaerobic threshold (AnT)–corresponding to the second lactate (LT2) or ventilatory threshold (VT2)–defines the transition from heavy to severe intensity, where lactate and V̇O_2_ rise progressively (Jamnick et al., 2020; Seiler, 2010; Stöggl & Sperlich, 2015). However, these conventional approaches are expensive, require specialized personnel and equipment, and are restricted to laboratory environments. Furthermore, blood lactate and gas exchange measurements reflect responses of specific physiological subsystems, whereas a more systemic approach to threshold determination may provide additional valuable insights (Gronwald et al., 2020; Rogers & Gronwald, 2022).

Recently, the short‐term scaling component alpha 1 (a1) derived from detrended fluctuation analysis (DFAa1) of heart rate (HR) variability (HRV) has been proposed as a practical, non‐invasive marker for exercise threshold determination (Gronwald et al., 2020; Gronwald & Hoos, 2020; Rogers & Gronwald, 2022). This non‐linear, dimensionless index reflecting the fractal correlation properties of beat‐to‐beat intervals is primarily influenced by parasympathetic and sympathetic modulation of sinoatrial node activity (Gronwald et al., 2020; Gronwald & Hoos, 2020; Hautala et al., 2003; Hottenrott, 2017; Michael et al., 2017; Peng et al., 1995; Rogers & Gronwald, 2022). Importantly, DFAa1 operates relatively independently from HR but demonstrates associations with oxygen uptake (V̇O_2_) and power output (PO), making it a promising candidate for exercise intensity demarcation (Rogers et al., 2020). At rest, healthy individuals typically exhibit DFAa1 values near 1.0, indicative of well‐correlated fractal behavior (Gronwald et al., 2020; Huikuri et al., 2000; Rogers & Gronwald, 2022). During incremental exercise, a characteristic biphasic pattern emerges: DFAa1 initially increases slightly above resting values at low‐to‐moderate exercise intensities due to parasympathetic withdrawal, then declines below 1.0 when transitioning to the heavy domain, and ultimately reaches ≤0.5 during severe exercise, reflecting loss of fractal complexity and subsystem disintegration (Gronwald et al., 2019, 2020; Gronwald & Hoos, 2020; Hautala et al., 2003; Rogers & Gronwald, 2022). In conclusion, a near‐linear drop of DFAa1 values from above 1.0 to below 0.5 can be observed when transitioning across intensity domains, highlighting its potential use in incremental exercise testing and intensity monitoring (Gronwald et al., 2019, 2020; Gronwald & Hoos, 2020; Rogers & Gronwald, 2022).

Specifically, two HRV thresholds (HRVT) have been proposed using standardized DFAa1 values: HRVT1 at DFAa1 = 0.75—the midpoint between well‐correlated (1.0) and uncorrelated (0.5) states‐putatively corresponding to VT1/LT1 (Gronwald et al., 2019; Hautala et al., 2003; Hottenrott, 2017; Michael et al., 2017); and HRVT2 at DFAa1 = 0.5, corresponding to VT2/LT2 (Fleitas‐Paniagua et al., 2024; Mateo‐March et al., 2023; Rogers et al., 2021; Schaffarczyk et al., 2023; Sempere‐Ruiz et al., 2024; Sheoran et al., 2024). Emerging evidence supports the validity of these thresholds across populations and exercise modalities (Held et al., 2025; Mateo‐March et al., 2023; Schaffarczyk et al., 2023; Sempere‐Ruiz et al., 2024; Sheoran et al., 2024). Additionally, a personalized approach (HRVT1_pers_) has been suggested to enhance accuracy by defining HRVT1 as the midpoint between the onset of the near‐linear DFAa1 decline and 0.5, reportedly yielding superior agreement with VT1 compared with the fixed 0.75 threshold (Rogers et al., 2024).

Taken together, DFAa1 is a non‐invasive and easy‐to‐use marker revealing potential for exercise threshold determination. Despite these promising developments, several critical knowledge gaps remain. First, few studies have simultaneously incorporated both gas exchange and blood lactate measurements when validating HRVTs using an incremental step protocol (Fleitas‐Paniagua et al., 2024; Kaufmann et al., 2023; Sempere‐Ruiz et al., 2024; Sheoran et al., 2024). Among these studies, methodological limitations constrain the generalizability of findings. Fleitas‐Paniagua et al. (2024) employed 2‐min incremental steps, shorter than recommended for reliable lactate threshold identification (Bentley et al., 2007; Jamnick et al., 2020), while both Sempere‐Ruiz et al. (2024) and Sheoran et al. (2024) used 3‐min steps but included only untrained participants. Notably, no investigation to date has examined HRVT validity using step durations exceeding 3 min, despite the widespread use of protocols with stages lasting 4–8 min in practical settings (Bentley et al., 2007, Jamnick et al., 2020). Given that step duration can substantially influence intensity zone demarcation (Bentley et al., 2007, Jamnick et al., 2020), evaluating HRVT validity in protocols with longer step durations is of considerable practical importance. Second, HRVT1_pers_ proposed by Rogers et al. (2024) has not yet been independently validated against lactate thresholds. Because this personalized method was applied exclusively for a ramp protocol with comparison to VT1, it remains unknown whether similar improvements in agreement occur within incremental step protocols or whether this approach alters the concordance between HRVT1 and LT1. Taken together, these gaps highlight the necessity to validate HRVTs in frequently used long‐step protocols reflective of practical settings and independently evaluate the innovative HRVT1_pers_ against the well‐established LT1. Hence, the *aim of this study* is to evaluate the agreement between HRVTs (HRVT1, HRVT1_pers_, and HRVT2), LTs, and VTs in trained adults during a 4‐min incremental cycling step test. We hypothesize that (1) both HRVT1 methods would exhibit good agreement with conventional thresholds; (2) HRVT1_pers_ would show superior agreement with LT1 and VT1 compared with the fixed HRVT1 (0.75) method; and (3) HRVT2 would demonstrate good agreement with both LT2 and VT2.

## 2 MATERIALS AND METHODS

### 2.1 Participants

Twenty‐four physically active male volunteers were recruited for this study. All participants were free from any cardiac arrhythmias, musculoskeletal injuries (<3 months), or recent illness (<1 month). Additionally, all participants engaged in at least 5 h of sports per week, were non‐smokers, and refrained from excessive alcohol consumption. Eventually, three participants were excluded due to inability to assess LTs correctly with the chosen methods for threshold determination (*n* = 1), illness (*n* = 1), and insufficient physical fitness levels (*n* = 1) (defined as LT1 PO <100 W). As such, data from twenty‐one participants were included for further analysis (Table 1). Sample size was determined a‐priori based on both sample sizes in previous studies (Rogers et al., 2020; Rogers et al., 2022; Van Hooren, Mennen, et al., 2023) and sample size calculations in G*Power (version 3.1.9.6). Estimated sample size varied between 12 and 21 participants (alpha‐level = 0.05, power = 0.8). Participants were instructed to avoid intense exercise for 36 h, alcohol consumption for 24 h, caffeine intake for at least 6 h, and heavy meals for 2 h prior to the tests. All sessions were conducted in the Bakala Athletic Performance Facility in Leuven, Belgium. The study protocol was approved by the Ethics Committee of UZ Leuven (S69345), and all participants provided written informed consent before participation. All procedures adhered to the principles of the Declaration of Helsinki.

**TABLE 1:** Descriptive statistics (mean ± standard deviation).

	*n* = 21
Age (years)	22.62 ± 3.44
Weight (kg)	71.44 ± 7.04
Height (m)	1.81 ± 0.06
Sports/Week (h)	10.1 ± 4.5
VO_2_peak (mL/kg/min)	55.8 ± 6.0

### 2.2 Maximal incremental exercise test

A stepwise maximal incremental protocol with 4‐min stages was conducted to determine exercise thresholds. The protocol started at 40W and increased by 30W at every stage. Participants were instructed to maintain a cycling cadence near 90 rpm. The incremental exercise test was terminated when a participant reached volitional exhaustion or cadence dropped below 80 rpm for more than 10 consecutive seconds. Strong verbal encouragement was provided during the test to ensure maximal effort.

### 2.3 Data collection

#### 2.3.1 Ventilatory and gas exchange measurements

Gas exchange variables were continuously measured throughout the incremental exercise test using a breath‐by‐breath metabolic analyzer (Metalyzer 3B, Cortex Biophysik, Leipzig, Germany). Prior to each test, the metabolic analyzer was calibrated according to the manufacturer's guidelines. Volume calibration was performed using a 3L syringe. Gas calibration was conducted using a known gas mixture (16.98% O_2_, 5.04% CO_2_, balance N_2_) and ambient air. Volume measurements were performed using a turbine flow sensor (Cortex Biophysik, Leipzig, Germany); oxygen concentrations were determined using a chemical fuel cell (Teledyne, CA, USA), and carbon dioxide concentrations were measured using a non‐dispersive infrared sensor (Treymed, NJ, USA). All tests were conducted under consistent environmental conditions (temperature: ~17.5°C, humidity: 50%–55%, ambient air composition: 20.93% O_2_, 0.03% CO_2_).

#### 2.3.2 Lactate measurements

Capillary blood lactate was measured after each 4‐min step using the Lactate Pro 2 analyzer (Arkray, Amstelveen, The Netherlands) from a 0.3 μL blood sample collected from the earlobe. When the first rise in lactate concentration was observed, a second blood sample was obtained to ensure accurate determination of LT1. Additionally, blood lactate measurements were performed immediately after test completion and 2 min post‐test.

#### 2.3.3 HR/RR measurements

HR and RR time series were continuously recorded during the incremental exercise test with a Polar H10 sensor (Polar Electro Oy, Kempele, Finland, sampling rate: 1000 Hz). HR sensor was standardized for each participant across study visits. HR belt electrodes were moistened to improve conductivity. To ensure accurate signal detection and adequate DFAa1 values, FatMaxxer (Android available, https://github.com/IanPeake/FatMaxxer) was used to verify signal quality prior to the test. Sufficient signal quality was based on R‐peak voltage (>1000 uV) according to prior recommendations (Rogers, 2022). One participant required repositioning of the HR sensor (1 cm to the left from the central position) to achieve sufficient signal quality (Rogers, 2022). HR belt positioning for all participants was standardized across visits.

The HR sensor was Bluetooth‐paired with a Polar Pacer watch, which was subsequently connected to Polar Flow. RR interval data was downloaded from Polar Flow as a.csv file and imported into Kubios HRV Premium (version 4.1.1, Biosignal Analysis and Medical Imaging Group, Department of Physics, University of Kuopio, Kuopio) for further analysis.

#### 2.3.4 Rate of perceived exertion (RPE)

RPE was assessed using a Borg scale ranging from 6 to 20 (Borg, 1982) at the end of each stage, immediately following lactate measurements.

### 2.4 Data analysis / threshold assessment

All threshold assessments were conducted under blinded conditions with respect to other physiological variables. To further reduce potential assessment bias, VT's were evaluated by examiners independent from those determining HRVT's.

#### 2.4.1 Ventilatory threshold assessment

Ventilatory data was extracted from the metabolic cart using a 30‐s moving average window. Ventilatory thresholds were assessed from the averaged data through an online platform (exphyslab.com (Keir et al., 2022)). VT1 was identified as the point where V̇CO_2_ and V̇ E increased disproportionately relative to V̇O_2_. VT1 was verified by steepening of the RER vs. V̇O_2_ curve, a systematic rise in V̇E/V̇O_2_ and PetO_2_ versus V̇O_2_, and the onset of a relatively stable isocapnic buffering phase, observed as stability in PetCO_2_ and V̇E/V̇CO_2_ versus V̇O_2_. All variables were considered to correctly define VT1 (Keir et al., 2022).

VT2 was identified by a rise in V̇E/V̇CO_2_ and the presence of a second breakpoint in the V̇E versus V̇O_2_ relationship. VT2 was verified by the end of stability in V̇E/V̇CO_2_ versus V̇O_2_, a steeper increase in V̇E/V̇O_2_ versus V̇O_2_, and the termination of the isocapnic buffering phase, marked by a decline in PetCO_2_ and a systematic increase in PetO_2_ (Keir et al., 2022).

Two independent examinators determined VTs twice on separate days. In case of an interrater difference in determined VTs >100 mL/min and an inability to reach consensus, a third independent assessor provided additional evaluation. VTs were then discussed until consensus was reached.

**FIGURE 1:**
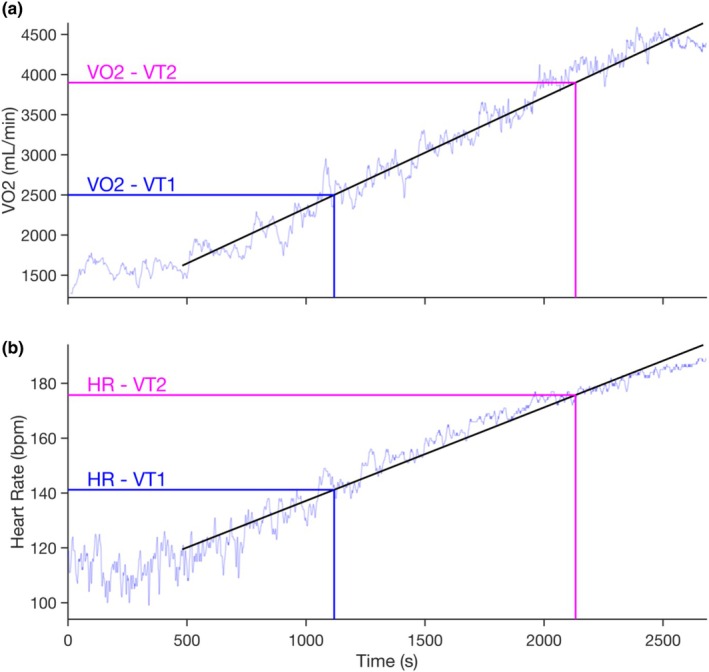
Visual representation of heart rate (HR) determination at the first (VT1) and second (VT2) ventilatory thresholds for one representative participant. (a) In the upper panel, a linear regression was performed between oxygen consumption (V̇O_2_) and time to determine the time corresponding to a given V̇O_2_. (b) In the lower panel, a second linear regression was conducted between time and HR to determine the HR at the previously calculated time points. The initial 480 s were excluded from both regression analyses as these were considered as warm‐up.

A linear regression of V̇O_2_ and time was performed to determine the timepoints at which the VTs occurred (Figure 1a). These timepoints were consequently used to estimate the corresponding PO and HR (Figure 1b). The first two steps (40–70 W) were excluded from data analysis and not included in the linear regression, since the ergometer used could not accurately measure PO in this range. Linear interpolation was used to convert stepwise increments in PO values to linearly increasing PO values. Given the step power increment of 30 W every 4 min, the linearly interpolated rate of power increment was 0.125 W/s (30 W/240 s = 0.125 W/s). HR at VTs was determined using a linear regression of HR versus Time (Figure 1b). VO_2_peak was defined as the highest value from the 30s moving average data.

#### 2.4.2 Lactate threshold assessment

**FIGURE 2:**
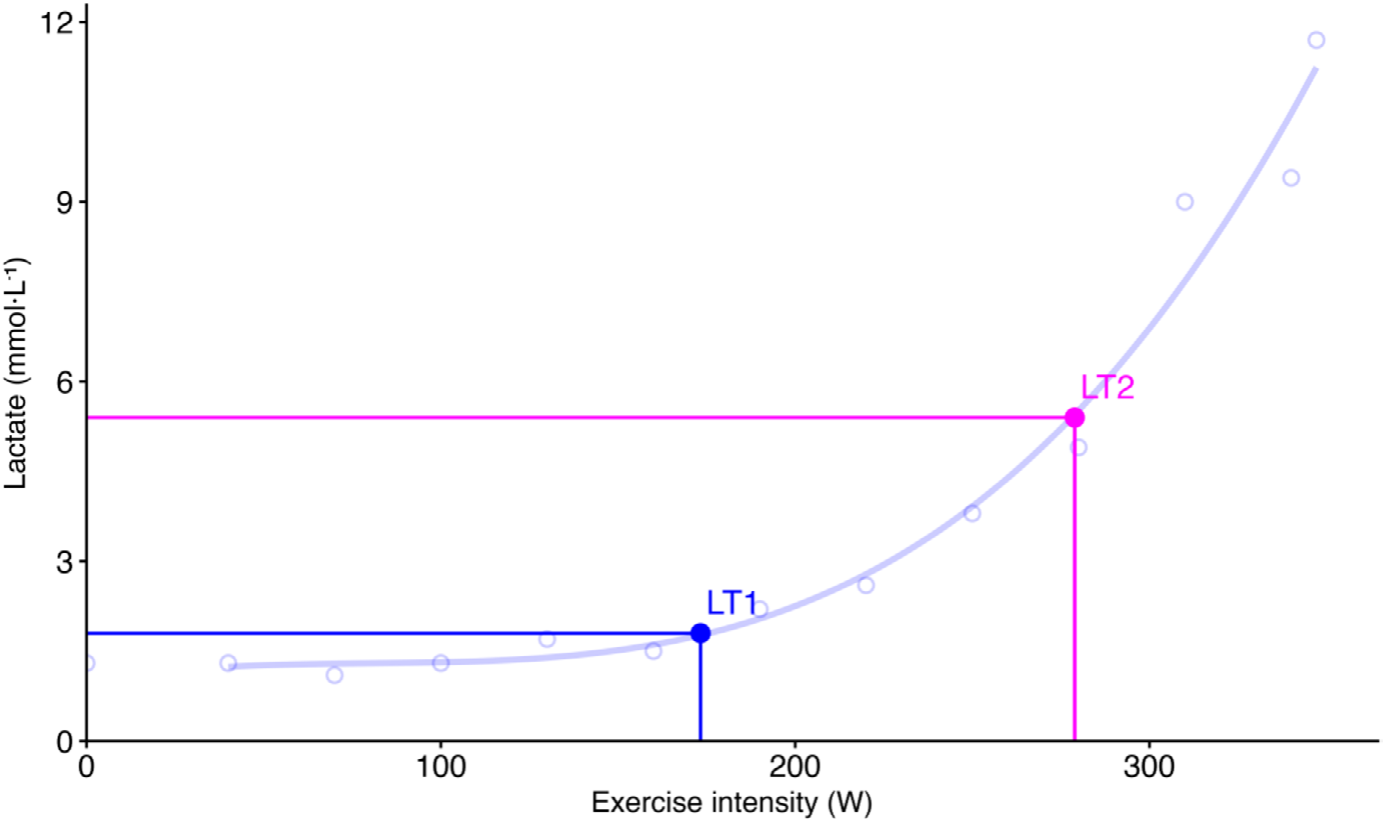
Example of lactate threshold determination for one representative participant. Baseline (Bsln) + 0.5 represents the first lactate threshold (LT1); Log‐Poly‐ModDmax represents the second lactate threshold (LT2). This figure was created using Rstudio code downloaded from exphyslab.com.

Lactate values were plotted using a third‐degree polynomial regression via exphyslab.com (Figure 2). LT1 was defined as the point at which blood lactate concentration increased by 0.5 mmol/L above baseline (Fleitas‐Paniagua et al., 2024). LT2 was determined using the Log–Log Modified DMax (Log‐Poly‐ModDMax) method. This method identifies LT2 as the point on the third‐degree polynomial curve with the greatest perpendicular distance to the straight line connecting the maximal lactate value and the log–log LT1 (Jamnick et al., 2018). This method was shown to provide the most accurate estimation of the MLSS from lactate values measured during a 4‐min protocol in trained cyclists (Jamnick et al., 2018).

The PO corresponding to LT1 and LT2 was converted into timepoints based on the linear interpolation of PO over time. These timepoints were used to obtain HR at LT1 and LT2 through the linear regression from HR over time, following the previously explained approach.

#### 2.4.3 Heart rate variability threshold assessment

RR‐intervals derived from the exercise test were imported into Kubios HRV Premium Software (version 4.1.1, Biosignal Analysis and Medical Imaging Group, Department of Physics, University of Kuopio, Kuopio). RR detrending method was set as “Smoothness priors” with a smoothing parameter of 500 and a cutoff frequency of 0.035 Hz. DFAa1 short‐term fluctuation window width was set to 4 ≤ *n* ≤16 beats (Tarvainen et al., 2014). The Kubios automatic beat correction was applied. The maximum acceptable artifact level was set at 5%, but no participants were excluded based on artifact levels (all <5%). DFAa1 values were computed every 5 s, using a 2‐min moving window. To calculate DFAa1 values, the root mean square fluctuation of integrated and detrended beat‐to‐beat interval time series data is measured in observation windows of different sizes and plotted against the size of the window on a log–log scale (Mendonca et al., 2010).

**FIGURE 3:**
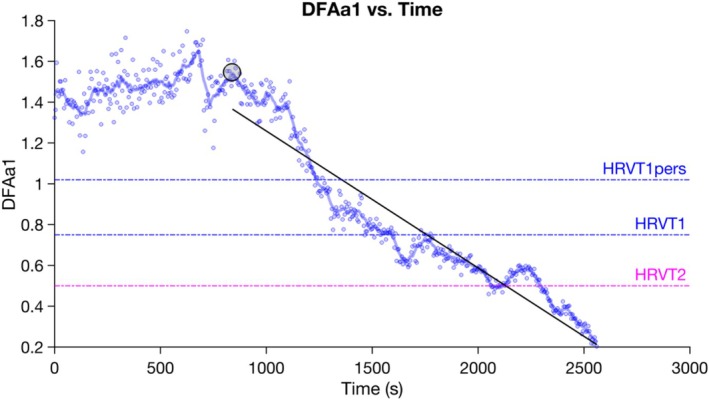
Visual representation of DFAa1 values during a maximal incremental exercise test for one representative participant. Determination of timepoints at the first (HRVT1), second (HRVT2), and first personalized (HRVT1_pers_) heart rate variability thresholds: HRVT1 (DFAa1 = 0.75), HRVT2 (DFAa1 = 0.50), and HRVT1_pers_ (DFAa1 = 1.02) are illustrated with horizontal lines. Max. DFAa1_start_ is annotated with a black circle and has a value of 1.54 for this participant.

The obtained DFAa1 values were then plotted over time in MATLAB R2022b (Version: 9.13.0.2049777). A linear regression was plotted for DFAa1 over time from the start of the near‐linear drop in DFAa1 values until the final timepoint (Figure 3). HRVT1 was defined as the point where the linear regression line crossed a DFAa1 value of 0.75 (Mateo‐March et al., 2023; Rogers et al., 2022; Schaffarczyk et al., 2023; Sempere‐Ruiz et al., 2024; Sheoran et al., 2024; Van Hooren, Mennen, et al., 2023). Additionally, and following the more recent methodology outlined by Rogers (Rogers et al., 2020), HRVT1_pers_ was determined as the midpoint between the highest DFAa1 value at the start of the linear drop (max. DFAa1_start_) and a DFAa1 value of 0.5, based on the following calculation: HRVT1_pers_ = (max. DFAa1_start_ + 0.5)/2 (Rogers et al., 2024). HRVT2 was defined as the point where the linear regression crossed a DFAa1 value of 0.5 (Fleitas‐Paniagua et al., 2024; Mateo‐March et al., 2023; Rogers et al., 2021; Schaffarczyk et al., 2023; Sempere‐Ruiz et al., 2024; Sheoran et al., 2024). Using the obtained time points, the previously described approach was used to determine the corresponding PO and HR at each HRVT.

### 2.5 Statistics

All variables‐VT1 HR/PO/V̇O_2_; LT1 HR/PO/V̇O_2_; HRVT1 HR/PO/V̇O_2_; HRVT1_pers_ HR/PO/V̇O_2_; VT2 HR/PO/V̇O_2_; LT2 HR/PO/V̇O_2_; and HRVT2 HR/PO/V̇O_2_‐were analyzed using Microsoft Excel 365 and RStudio (version 4.3.1, 2023‐06‐16).

Normality of all variables for threshold determination was assessed using the Shapiro–Wilk's test. A one‐way repeated measures analysis of variance (ANOVA) was used for all normally distributed variables. For the non‐normally distributed pair (LT1 PO vs. HRVT1 PO), the Friedman test was applied. Bonferroni post hoc corrections were used to account for multiple comparisons. The relationships and agreements between variables were assessed using the Intraclass Correlation Coefficient (ICC2,1), Pearson's correlation coefficient for normally distributed data, and Spearman's rank correlation coefficient for non‐normally distributed data. Bland–Altman plots with 95% limits of agreement (LOA) were used to evaluate agreement between methods of threshold determination.

Pearson correlations were interpreted as trivial (<0.10), small (0.10–0.29), moderate (0.30–0.49), large (0.50–0.69), very large (0.70–0.89), and nearly perfect (0.90–0.99) (Hopkins). ICC was evaluated as poor (<0.50), moderate (0.5–0.74), good (0.75–0.90), and excellent (>0.90) (Koo & Li, 2016). Statistical significance was set at *p* < 0.05 for all analyses.

## 3 RESULTS

All variables were normally distributed (*p* > 0.05) except for the difference in LT1 PO versus HRVT1 PO (*p* = 0.026). Descriptive statistics are reported as mean ± standard deviation (SD) in Table 2. Comparisons for HRVT1_(pers)_ versus VT1 versus LT1 are reported in Table 3 and comparisons for HRVT2 versus VT2 versus LT2 are reported in Table 4. Bland–Altman plots with LOA are provided in Supplementary Materials (S1 and S2).

**TABLE 2:** Mean ± standard deviation of HR, PO and V̇O_2_ at VT1, LT1, HRVT1, HRVT1_pers_, VT2, LT2, and HRVT2.

	HR (bpm)	PO (W)	V̇O_2_ (mL/kg/min)
VT1	144 ± 10	156 ± 18	34 ± 4
LT1	153 ± 14	180 ± 37	38 ± 6
HRVT1	161 ± 11	201 ± 34	40 ± 5
HRVT1_pers_	153 ± 11	177 ± 33	37 ± 5
VT2	166 ± 09	226 ± 29	45 ± 5
LT2	172 ± 11	234 ± 33	46 ± 5
HRVT2	173 ± 10	236 ± 33	46 ± 5

Abbreviations: HR, heart rate; HRVT1, first heart rate variability threshold; HRVT1_pers_, first personalized heart rate variability threshold; HRVT2, second heart rate variability threshold; LT1, first lactate threshold; LT2, second lactate threshold; PO, power output; V̇O_2_, oxygen uptake; VT1, first ventilatory threshold; VT2, second ventilatory threshold.

**TABLE 3:** Comparison of HRVT1_(pers)_ versus VT1 versus LT1. The table is read from top (columns) to left (rows).

VT1								LT1							
		ICC	Mean bias	Lower LOA	Upper LOA	*r*‐value	ANOVA			ICC	Mean bias	Lower LOA	Upper LOA	*r*‐value	ANOVA
HR	LT1	0.53	−9	−30	11	0.51*	0.002	HR	VT1	0.53	9	−11	30	0.51*	0.002*
	HRVT1	0.16	−17	−41	7	0.36	<0.001*		HRVT1	0.30	−8	−36	21	0.07	0.150
	HRVT1_pers_	0.26	−9	−33	15	0.35	0.020*		HRVT1_pers_	0.30	0	−29	30	0.03	1.000
PO	LT1	0.40	−24	−81	33	0.65*	0.008*	PO	VT1	0.40	24	−33	81	0.65*	0.008*
	HRVT1	0.07	−45	−117	27	0.18	<0.001*		HRVT1	0.33	−21	−100	58	0.38	0.174
	HRVT1_pers_	0.08	−21	−93	50	0.10	0.048*		HRVT1_pers_	0.30	3	−80	86	0.18	1.000
V̇O_2_	LT1	0.52	−4	−12	5	0.72**	0.008*	V̇O_2_	VT1	0.52	4	−5	12	0.72**	0.008*
	HRVT1	0.16	−6	−17	6	0.29	0.001*		HRVT1	0.35	−2	−15	11	0.37	1.000
	HRVT1_pers_	0.28	−3	−14	8	0.35	0.109		HRVT1_pers_	0.32	0	−13	14	0.32	1.000

Abbreviations: ANOVA, analysis of variance; HR, heart rate; HRVT1, first heart rate variability threshold; HRVT1_pers_, first personalized heart rate variability threshold; ICC, intraclass correlation; LOA, limits of agreement; LT1, first lactate threshold; PO, power output; VO_2_, oxygen uptake; VT1, first ventilatory threshold.

* *p* < 0.05.

** *p* < 0.001.

**TABLE 4:** Comparison of HRVT2 versus VT2 versus LT2. The table is read from top (columns) to left (rows).

VT2								LT2							
		ICC	Mean bias	Lower LOA	Upper LOA	Pearson	ANOVA			ICC	Mean bias	Lower LOA	Upper LOA	*r*‐value	ANOVA
HR	LT2	0.69	−6	−18	7	0.64*	0.001*	HR	VT2	0.69	6	−7	18	0.64*	0.001*
	HRVT2	0.47	−6	−24	11	0.50*	0.009*		HRVT2	0.65	−1	−18	16	0.48**	1.000
PO	LT2	0.84	−8	−41	24	0.87**	0.096	PO	VT2	0.84	8	−24	41	0.87*	0.096*
	HRVT2	0.65	−10	−60	40	0.68**	0.257		HRVT2	0.68	−2	−56	52	0.67	1.000
V̇O_2_	LT2	0.84	−1	−6	4	0.89**	0.121	V̇O_2_	VT2	0.84	1	−4	6	0.89**	0.121
	HRVT2	0.65	−1	−9	6	0.73**	0.339		HRVT2	0.68	0	−9	8	0.68**	1.000

Abbreviations: ANOVA, analysis of variance; HR, heart rate; HRVT2, second heart rate variability threshold; ICC, intraclass correlation; LOA, limits of agreement; LT2, second lactate threshold; PO, power output; VO_2_, oxygen uptake; VT2, second ventilatory threshold.

* *p* < 0.05.

** *p* < 0.001.

### 3.1 VT1 and LT1 versus HRVT1

Poor ICC values (0.07–0.35) were found for both VT1 versus HRVT1 and LT1 versus HRVT1 across all measured parameters (HR, PO, V̇O_2_). Similarly, correlation coefficients indicated trivial to moderate associations (0.07–0.37). Bonferroni‐corrected pairwise comparisons revealed significant mean differences for VT1 versus HRVT1 for all variables, while no significant differences between LT1 and HRVT1 were found. Bland–Altman analyses showed substantial mean biases and wide LOA across all variables when comparing VT1 versus HRVT1 (−17 ± 24 bpm for HR; −45 ± 72 W for PO and − 6 ± 11 mL/kg/min for V̇O_2_) or LT1 versus HRVT1 (−8 ± 28 bpm for HR; −21 ± 79 W for PO and −2 ± 13 mL/kg/min for V̇O_2_).

### 3.2 VT1 and LT1 versus HRVT1_pers_

HRVT1_pers_ exhibited poor ICC (0.08–0.33) and trivial to moderate (0.03–0.35) correlation coefficients when compared to VT1 and LT1. ANOVA revealed significant differences for HR and PO between VT1 and HRVT1_pers_, whereas no mean differences between LT1 and HRVT1_pers_ were observed. Bland–Altman plots for VT1 and HRVT1_pers_ showed large mean differences and wide LOA (−9 ± 24 bpm for HR; −21 W ± 72 W for power and − 3 ± 11 mL/kg/min for V̇O_2_). For LT1 versus HRVT1_pers_, mean differences were minimal, albeit with wide LOA (0 ± 30 bpm for HR; 3 ± 83 W for PO and 0 ± 14 mL/kg/min for V̇O_2_).

### 3.3 VT2 and LT2 versus HRVT2

Overall, agreements between VT2 or LT2 versus HRVT2 were found to be more congruent to agreements revealed for VT1 or LT1 versus HRVT1. ICC values were moderate (0.65–0.68) for all parameters except HR, with poor ICC (0.47) between VT2 HR and HRVT2 HR. This was supported by a statistically significant difference for HR at these thresholds (*p* < 0.05). Correlation coefficients ranged from large to very large (0.50–0.73), except for LT2 HR vs. HRVT2 HR, which showed a moderate correlation (0.48). Bland–Altman indicated small to negligible mean biases but relatively wide LOA for VT2 versus HRVT2 (−6 ± 17 bpm for HR; −10 ± 50 W for PO and −1 ± 7 mL/kg/min for V̇O_2_) and LT2 versus HRVT2 (−1 ± 17 bpm for HR; −2 ± 54 W for PO and 0 ± 8 mL/kg/min for V̇O_2_).

## 4 DISCUSSION

The *aim* of the present study was to examine the agreement between DFAa1‐derived HRV thresholds and gold‐standard threshold determination techniques during a 4‐min stepwise incremental cycling protocol. The principal findings were threefold: (1) HRVT1 demonstrated poor agreement with VT1/LT1 across all measured variables (HR, PO, V̇O_2_), with substantial systematic bias; (2) HRVT1_pers_ approach yielded only marginal improvements over the fixed 0.75 threshold; and (3) HRVT2 showed considerably stronger agreement with VT2/LT2, particularly for PO and V̇O_2_, although wide LOA persisted. These findings are at odds with our first two hypotheses, while confirming the third. Notably, they are supported by the majority of recent investigations in this area (Fleitas‐Paniagua et al., 2024; Mateo‐March et al., 2023; Schaffarczyk et al., 2023; Sempere‐Ruiz et al., 2024).

### 4.1 Limited agreement between HRVT1 and aerobic threshold (Table 3)

HRVT1 exhibited poor agreement with both VT1 and LT1, characterized by weak correlations (*r* = 0.07–0.38), low ICCs (0.07–0.35), and substantial systematic bias across all variables (HR: −8 to −17 bpm; PO: −21 to −45 W; V̇O_2_: −2 to −6 mL/kg/min). HRVT1_pers_, despite theoretical advantages, yielded only marginal improvements in agreement (*r* = 0.03–0.35; ICC = 0.08–0.33) and failed to substantially reduce systematic bias. These findings suggest that the DFAa1 value of 0.75 may represent a more arbitrary construct– the mathematical midpoint between correlated (1.0) and uncorrelated (0.5) dynamics– rather than a distinct physiological transition (Fleitas‐Paniagua et al., 2024). The lack of improvement with the personalized approach is particularly noteworthy, as Rogers et al. (2024) reported enhanced agreement between HRVT1_pers_ and VT1 in a ramp protocol. This discrepancy suggests that the individualized method may be protocol‐dependent and less effective in stepwise protocols where stage duration influences DFAa1 kinetics.

### 4.2 Agreement between HRVT2 and anaerobic threshold (Table 4)

In contrast, the relatively strong agreement observed between HRVT2 and both LT2 and VT2– evidenced by moderate‐to‐strong correlations (*r* = 0.67–0.73) and ICCs (0.65–0.68)– suggests that the DFAa1 value of 0.5 represents a physiologically meaningful transition point. This threshold reflects a shift from uncorrelated to anticorrelated fractal behavior in HR dynamics, corresponding to a state of maximal cardiovascular self‐regulatory demand that can only be transiently sustained (Fleitas‐Paniagua et al., 2024; Gronwald et al., 2020). The small mean biases for PO (−2 to −10 W) and V̇O_2_ (−1 to 0 mL/kg/min) at HRVT2 further support its potential utility as a surrogate marker for the anaerobic threshold. These findings are consistent with previous work demonstrating good concordance between HRVT2 and VT2/LT2 across various protocols and populations (Mateo‐March et al., 2023; Schaffarczyk et al., 2023; Sempere‐Ruiz et al., 2024). However, the wide LOA observed for PO (50–54 W) and V̇O_2_ (7–9 mL/kg/min) indicate considerable individual variability and suggest that HRVT2 should be interpreted cautiously at the individual level, particularly when precise intensity prescription is required.

### 4.3 Sources of inter‐study variability

Despite the overall consistency of our findings with recent literature, some studies have reported superior agreement for HRVT1 (Mateo‐March et al., 2023; Rogers et al., 2022; Schaffarczyk et al., 2023; Sempere‐Ruiz et al., 2024). Several methodological and population‐related factors may account for these discrepancies.

#### 4.3.1 Protocol‐related factors

Testing protocol characteristics can substantially influence HRV, V̇O_2_, and lactate kinetics (Bentley et al., 2007; Jamnick et al., 2020). Although Fleitas‐Paniagua et al. (2023) reported no effect of ramp slope (15, 30, or 45 W/min) on HR or V̇O_2_ at HRVT1, emerging evidence indicates that DFAa1 is sensitive not only to exercise intensity but also to exercise duration (Rogers et al., 2025; van Rassel et al., 2025). The present study employed a stepwise protocol with relatively small power increments (equivalent to approximately 7.5 W/min; resulting in an average total duration of 38 min 35 s) rather than a continuous ramp (usually 10–15 min in total) (Fleitas‐Paniagua et al., 2023; Iannetta et al., 2020), which may have accentuated the influence of exercise duration on HRVT detection. Furthermore, the 4‐min stage duration used here– selected to ensure adequate time for lactate steady state (Jamnick et al., 2018, 2020)–exceeds that of all previous HRVT validation studies. While this longer duration may have improved LT accuracy, it could have been suboptimal for VT detection, as shorter stages are typically recommended for gas exchange threshold identification (Jamnick et al., 2020). This protocol‐dependent trade‐off may partially explain the observed discrepancies between HRVT1 and both VT1 and LT1.

#### 4.3.2 Methodological variability

Threshold determination methods introduce variability. Numerous lactate threshold identification techniques exist, each with distinct criteria and sensitivities (Jamnick et al., 2020). Similarly, ventilatory threshold identification remains inherently subjective despite standardized visual inspection criteria, introducing operator‐dependent variability (Kaczmarek et al., 2019). These inherent limitations of gold‐standard methods complicate the validation of novel approaches such as DFAa1‐based thresholds.

#### 4.3.3 Population characteristics

Although DFAa1 is a dimensionless index theoretically independent of sex, age, and fitness level, some evidence suggests population‐dependent variability. Sheoran et al. (2024) reported underestimation of thresholds in highly trained individuals and overestimation in less fit athletes, potentially attributable to slower homeostatic responses in lower‐fitness populations. However, our findings in trained participants do not clearly support this interpretation, as we generally observed an overestimation of HRVT. Additionally, hormonal fluctuations across the menstrual cycle have been shown to influence autonomic balance and DFAa1 behavior (Rogers et al., 2022; Schaffarczyk et al., 2023), and greater bias has been reported in males, possibly due to higher inter‐individual variability in fitness levels (Sheoran et al., 2024). Consequently, caution is warranted when extrapolating these results to other populations, and inter‐study variability may be partly attributable to differences in participant characteristics.

### 4.4 Limitations and future directions

While this study was the first to explore DFAa1 threshold determination in a 4‐min incremental cycling protocol, several limitations warrant acknowledgement. First, the inclusion of only trained male participants limits generalizability to female athletes and untrained populations. Second, while the 4‐min stage duration was selected for practical relevance and lactate‐steady state attainment, this may have compromised VT assessment accuracy (Jamnick et al., 2018, 2020). Third, further validation of HRVT1_pers_ against lactate thresholds is warranted, particularly in protocols with varying stage durations. Lastly, although previous work has shown DFAa1 to be reliable at a given exercise intensity (Sempere‐Ruiz et al., 2024; Sheoran et al., 2024; Van Hooren, Bongers, et al., 2023), the present study did not directly assess the test–retest reliability of DFAa1‐derived thresholds, nor their responsiveness to training interventions.

Beyond these methodological considerations, future research should focus on comparative studies employing multiple stage durations within the same participants to clarify the protocol‐dependency of HRVT validity. Another potential step forward involves leveraging the reliability of DFAa1 at a given exercise intensity (Sempere‐Ruiz et al., 2024; Sheoran et al., 2024; Van Hooren, Bongers, et al., 2023) for longitudinal monitoring. If an individual exhibits a personalized DFAa1 value of 0.85 at LT1 corresponding to 175 W in an initial test, and subsequently reaches this same DFAa1 value at 225 W following a training intervention, this may indicate meaningful improvement in aerobic capacity without requiring repeated laboratory testing.

### 4.5 Practical applications

From a practical standpoint, our findings suggest that DFAa1 may not be suitable for aerobic threshold determination in a 4‐min stepwise protocol, given the wide LOA and poor agreement with established methods. However, for anaerobic threshold assessment, HRVT2 could serve as a complementary marker alongside established gold‐standard techniques, particularly when laboratory testing is impractical or unavailable. Importantly, gold‐standard methods themselves exhibit variability and have limitations, often also demonstrating mean biases and wide LOA when compared with one another (Kaufmann et al., 2023; Pallarés et al., 2016; Tanner et al., 2024).

Moreover, as highlighted by Hoos and Gronwald, (2025), a mismatch between HRVT1 and VT1/LT1 does not necessarily invalidate either conventional methods or DFAa1 for exercise intensity prescription. Rather, emphasis should be placed on longitudinal monitoring, focusing on the translation of laboratory‐based thresholds to field‐based training, day‐to‐day intensity fine‐tuning, and optimization of training adaptations. Within this framework, DFAa1 may represent a practical, accessible, and user‐friendly tool for approximate exercise intensity assessment without requiring prior laboratory testing. The ability to monitor training intensity in real‐world settings using consumer‐grade HR monitors (Rogers et al., 2025) offers considerable practical advantages for athletes and coaches, despite the limitations in absolute threshold determination.

## 5 CONCLUSION

In conclusion, this study demonstrated poor agreement between HRVT1 and established aerobic threshold markers (VT1/LT1) in a 4‐min stepwise protocol, with only marginal improvement using the personalized HRVT1_pers_ approach. In contrast, HRVT2 showed good agreement with anaerobic threshold markers (VT2/LT2), particularly for power output and oxygen uptake, although wide limits of agreement indicate substantial individual variability. These findings suggest that DFAa1 at 0.5 may complement anaerobic threshold determination in a 4‐min incremental stage protocol. The practical utility of DFAa1 may lie not in replacing laboratory‐based threshold testing but in enabling accessible, longitudinal monitoring of training intensity, and adaptation in field settings.

## FUNDING INFORMATION

KU Leuven Special Fund (grant no. 3M220016).

## CONFLICT OF INTEREST STATEMENT

The authors declare no conflicts of interest.

## ETHICS STATEMENT

This study was a randomized controlled trial involving human participants. Ethical approval was obtained from the appropriate institutional ethics committee (Ethics Committee of UZ Leuven (S69345)) prior to the commencement of the study.

## CODE AVAILABILITY STATEMENT

The codes used for data processing and analysis are available from the corresponding author upon reasonable request.

## PATIENT CONSENT STATEMENT

Written informed consent was obtained from all participants before enrollment and prior to the start of the study.

## CLINICAL TRIAL REGISTRATION

This randomized controlled trial was registered with the Ethics Committee Research UZ/KU Leuven (Reference: S69345; Belgian Registration Number: B3222024001515) prior to the initiation of participant enrollment.

## Supporting information


**Figure S1.** (A) Bland–Altman plots of HRVT1_(pers)_ versus VT1 versus LT1 for HR. HR, heart rate; HRVT1, first heart rate variability threshold; HRVT1pers, first personalized heart rate variability threshold; LOA, limits of agreement; LT1, first lactate threshold; VT1, first ventilatory threshold. (B) Bland–Altman plots of HRVT1_(pers)_ versus VT1 versus LT1 for PO. HRVT1, first heart rate variability threshold; HRVT1pers, first personalized heart rate variability threshold; LOA, limits of agreement; LT1, first lactate threshold; PO, power output; VT1, first ventilatory threshold. (C) Bland–Altman plots of HRVT1_(pers)_ versus VT1 versus LT1 for VO_2_. HRVT1, first heart rate variability threshold; HRVT1pers, first personalized heart rate variability threshold; LOA, limits of agreement; LT1, first lactate threshold; VO_2_, oxygen uptake; VT1, first ventilatory threshold.
**Figure S2.** (A) Bland–Altman plots of HRVT2 versus VT2 versus LT2 for HR. HR, heart rate; HRVT2, second heart rate variability threshold; LOA, limits of agreement; LT2, second lactate threshold; VT2, second ventilatory threshold. (B) Bland–Altman plots of HRVT2 versus VT2 versus LT2 for PO. HRVT2, second heart rate variability threshold; LOA, limits of agreement; LT2, second lactate threshold; PO, power output; VT2, second ventilatory threshold. (C) Bland–Altman plots of HRVT2 versus VT2 versus LT2 for VO_2_. HR, heart rate; HRVT2, second heart rate variability threshold; LOA, limits of agreement; LT2, second lactate threshold; PO, power output; VO_2_, oxygen uptake; VT2, second ventilatory threshold.

## Data Availability

The datasets generated and/or analyzed during the current randomized controlled trial are available from the corresponding author upon reasonable request.
