# Supplementary figures and images for: Agreement between heart rate variability‐derived and lactate/ventilatory thresholds during a 4‐min stepwise incremental cycling test in male adults
Source: Physiol Rep. 2026 Feb 16;14(4):e70777. doi: 10.14814/phy2.70777 (PMC12910119; doi:10.14814/phy2.70777)

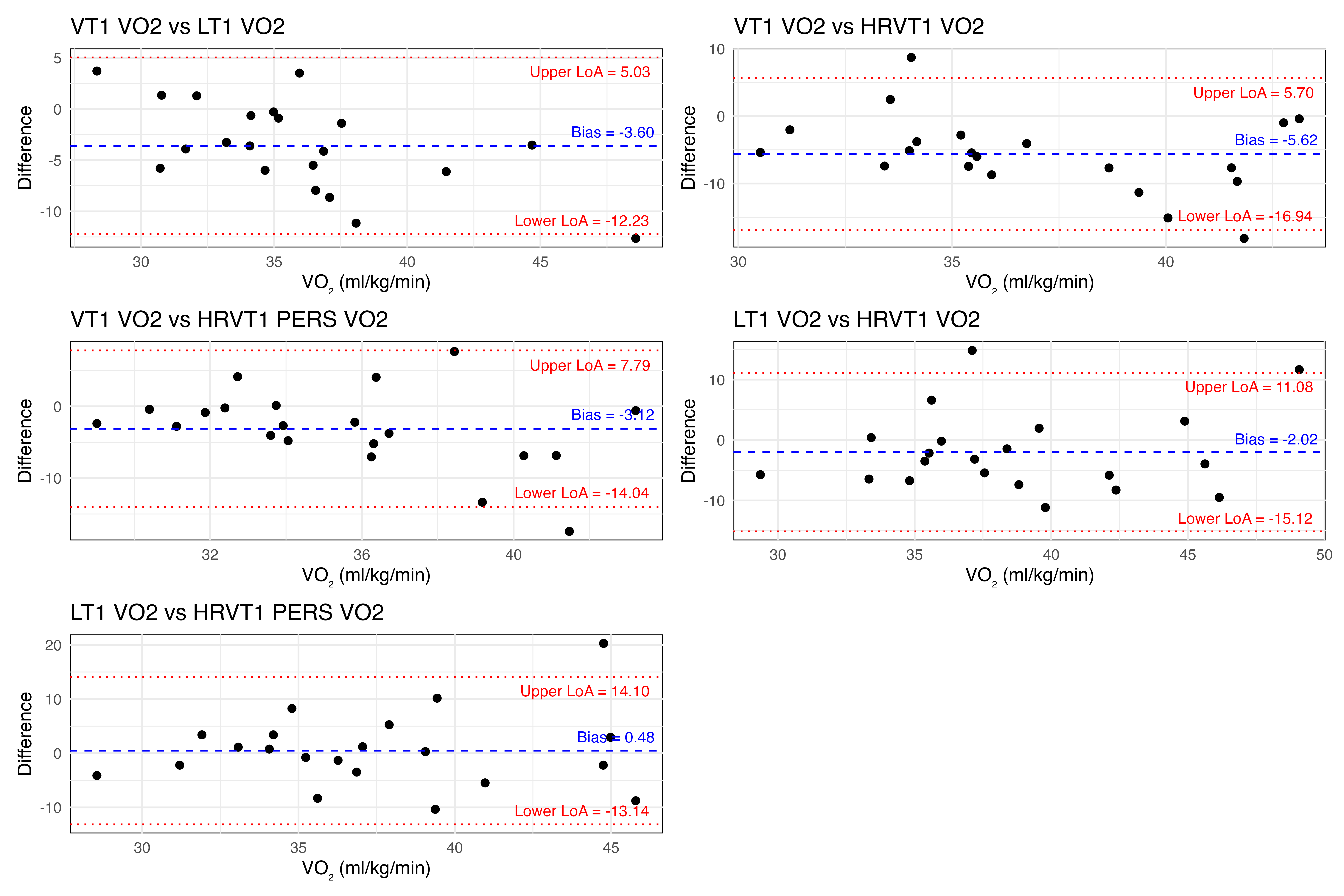

Supplement: Supplementary file 1 — Figure S1. (A) Bland–Altman plots of HRVT1(pers) versus VT1 versus LT1 for HR. HR, heart rate; HRVT1, first heart rate variability threshold; HRVT1pers, first personalized heart rate variability threshold; LOA, limits of agreement; LT1, first lactate threshold; VT1, first ventilatory threshold. (B) Bland–Altman plots of HRVT1(pers) versus VT1 versus LT1 for PO. HRVT1, first heart rate variability threshold; HRVT1pers, first personalized heart rate variability threshold; LOA, limits of agreement; LT1, first lactate threshold; PO, power output; VT1, first ventilatory threshold. (C) Bland–Altman plots of HRVT1(pers) versus VT1 versus LT1 for VO2. HRVT1, first heart rate variability threshold; HRVT1pers, first personalized heart rate variability threshold; LOA, limits of agreement; LT1, first lactate threshold; VO2, oxygen uptake; VT1, first ventilatory threshold. Figure S2. (A) Bland–Altman plots of HRVT2 versus VT2 versus LT2 for HR. HR, heart rate; HRVT2, second heart rate variability threshold; LOA, limits of agreement; LT2, second lactate threshold; VT2, second ventilatory threshold. (B) Bland–Altman plots of HRVT2 versus VT2 versus LT2 for PO. HRVT2, second heart rate variability threshold; LOA, limits of agreement; LT2, second lactate threshold; PO, power output; VT2, second ventilatory threshold. (C) Bland–Altman plots of HRVT2 versus VT2 versus LT2 for VO2. HR, heart rate; HRVT2, second heart rate variability threshold; LOA, limits of agreement; LT2, second lactate threshold; PO, power output; VO2, oxygen uptake; VT2, second ventilatory threshold. [file PHY2-14-e70777-s001.zip › PHYSREP-2025-12-1086-s03.png]

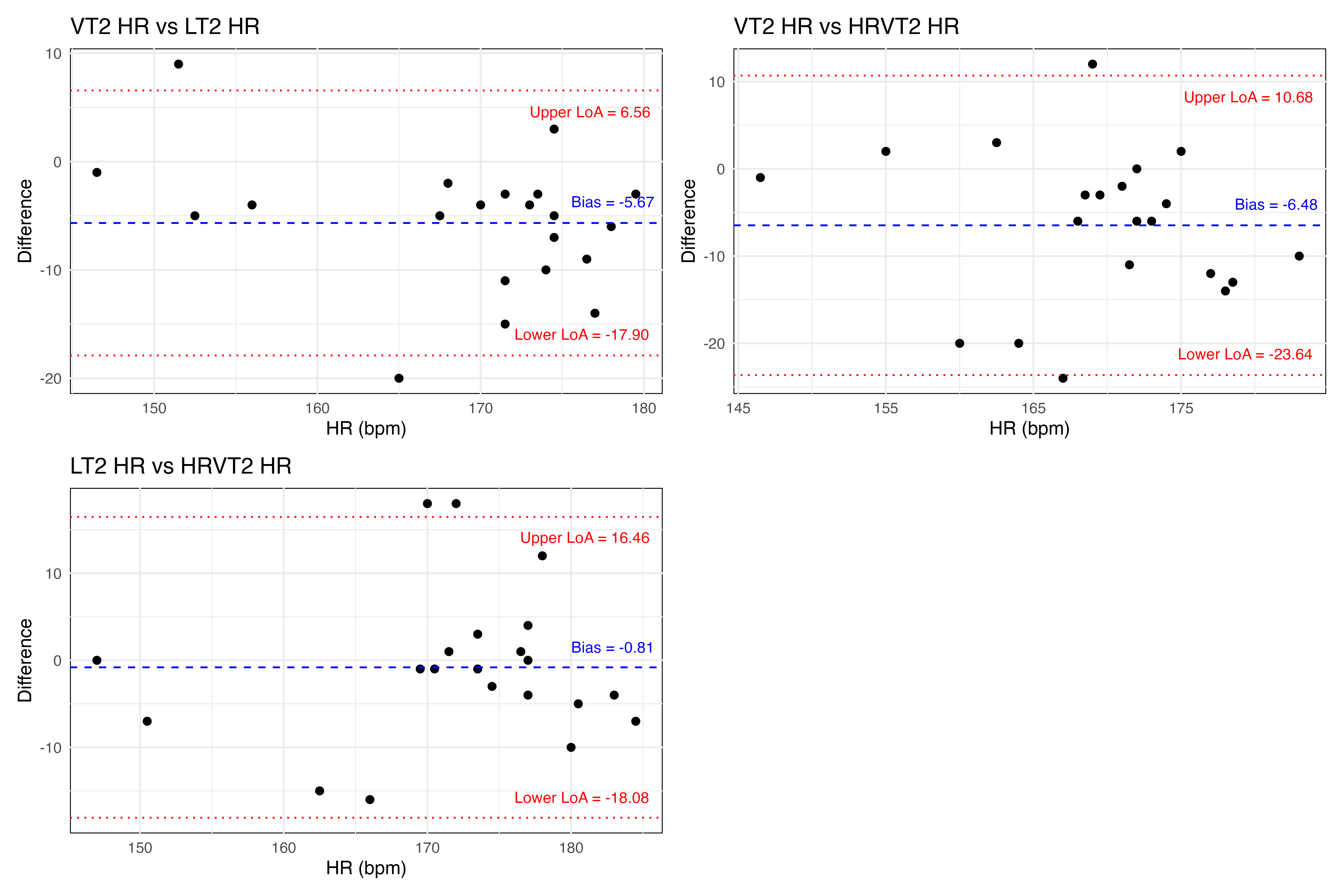

Supplement: Supplementary file 1 — Figure S1. (A) Bland–Altman plots of HRVT1(pers) versus VT1 versus LT1 for HR. HR, heart rate; HRVT1, first heart rate variability threshold; HRVT1pers, first personalized heart rate variability threshold; LOA, limits of agreement; LT1, first lactate threshold; VT1, first ventilatory threshold. (B) Bland–Altman plots of HRVT1(pers) versus VT1 versus LT1 for PO. HRVT1, first heart rate variability threshold; HRVT1pers, first personalized heart rate variability threshold; LOA, limits of agreement; LT1, first lactate threshold; PO, power output; VT1, first ventilatory threshold. (C) Bland–Altman plots of HRVT1(pers) versus VT1 versus LT1 for VO2. HRVT1, first heart rate variability threshold; HRVT1pers, first personalized heart rate variability threshold; LOA, limits of agreement; LT1, first lactate threshold; VO2, oxygen uptake; VT1, first ventilatory threshold. Figure S2. (A) Bland–Altman plots of HRVT2 versus VT2 versus LT2 for HR. HR, heart rate; HRVT2, second heart rate variability threshold; LOA, limits of agreement; LT2, second lactate threshold; VT2, second ventilatory threshold. (B) Bland–Altman plots of HRVT2 versus VT2 versus LT2 for PO. HRVT2, second heart rate variability threshold; LOA, limits of agreement; LT2, second lactate threshold; PO, power output; VT2, second ventilatory threshold. (C) Bland–Altman plots of HRVT2 versus VT2 versus LT2 for VO2. HR, heart rate; HRVT2, second heart rate variability threshold; LOA, limits of agreement; LT2, second lactate threshold; PO, power output; VO2, oxygen uptake; VT2, second ventilatory threshold. [file PHY2-14-e70777-s001.zip › PHYSREP-2025-12-1086-s04.png]

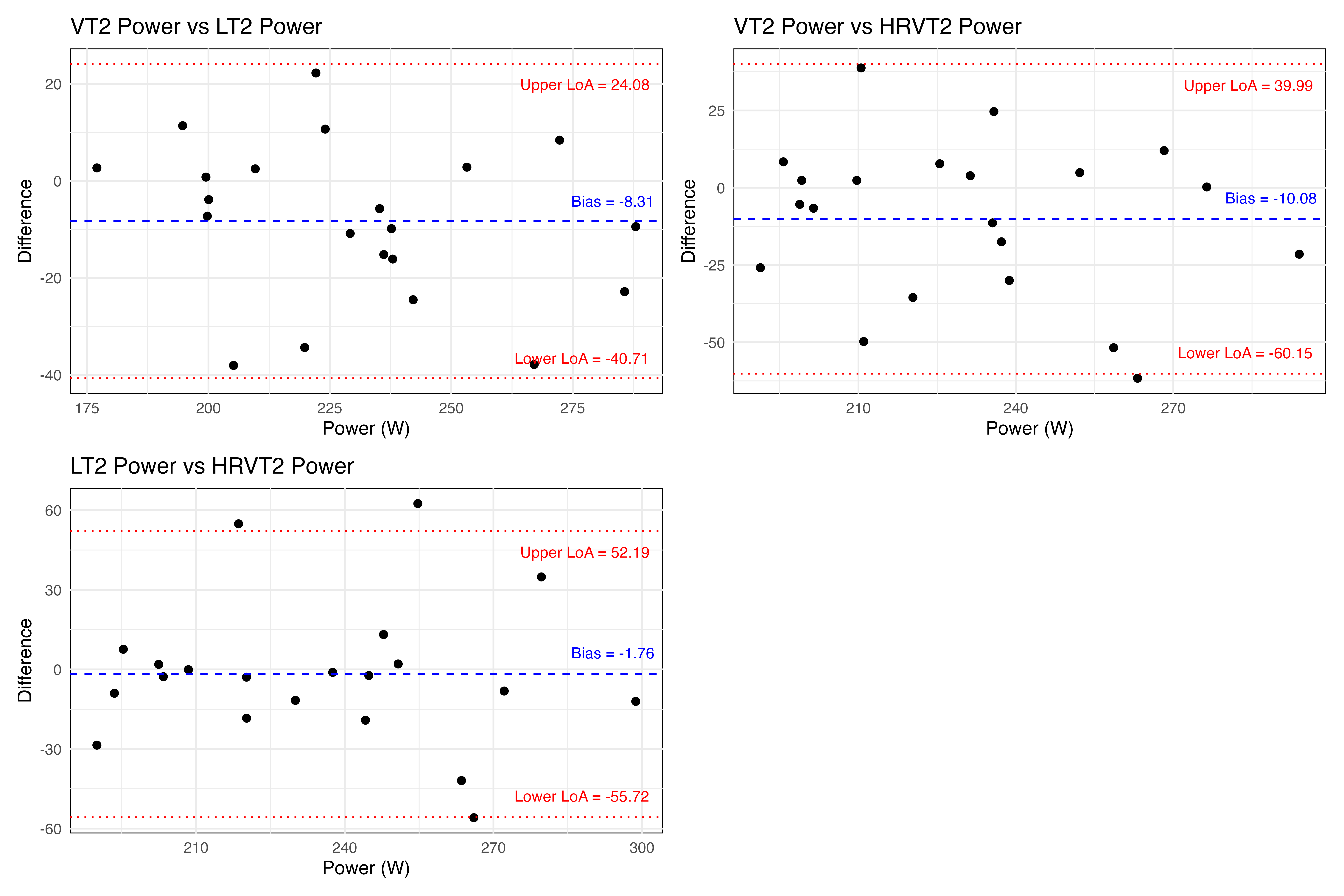

Supplement: Supplementary file 1 — Figure S1. (A) Bland–Altman plots of HRVT1(pers) versus VT1 versus LT1 for HR. HR, heart rate; HRVT1, first heart rate variability threshold; HRVT1pers, first personalized heart rate variability threshold; LOA, limits of agreement; LT1, first lactate threshold; VT1, first ventilatory threshold. (B) Bland–Altman plots of HRVT1(pers) versus VT1 versus LT1 for PO. HRVT1, first heart rate variability threshold; HRVT1pers, first personalized heart rate variability threshold; LOA, limits of agreement; LT1, first lactate threshold; PO, power output; VT1, first ventilatory threshold. (C) Bland–Altman plots of HRVT1(pers) versus VT1 versus LT1 for VO2. HRVT1, first heart rate variability threshold; HRVT1pers, first personalized heart rate variability threshold; LOA, limits of agreement; LT1, first lactate threshold; VO2, oxygen uptake; VT1, first ventilatory threshold. Figure S2. (A) Bland–Altman plots of HRVT2 versus VT2 versus LT2 for HR. HR, heart rate; HRVT2, second heart rate variability threshold; LOA, limits of agreement; LT2, second lactate threshold; VT2, second ventilatory threshold. (B) Bland–Altman plots of HRVT2 versus VT2 versus LT2 for PO. HRVT2, second heart rate variability threshold; LOA, limits of agreement; LT2, second lactate threshold; PO, power output; VT2, second ventilatory threshold. (C) Bland–Altman plots of HRVT2 versus VT2 versus LT2 for VO2. HR, heart rate; HRVT2, second heart rate variability threshold; LOA, limits of agreement; LT2, second lactate threshold; PO, power output; VO2, oxygen uptake; VT2, second ventilatory threshold. [file PHY2-14-e70777-s001.zip › PHYSREP-2025-12-1086-s05.png]

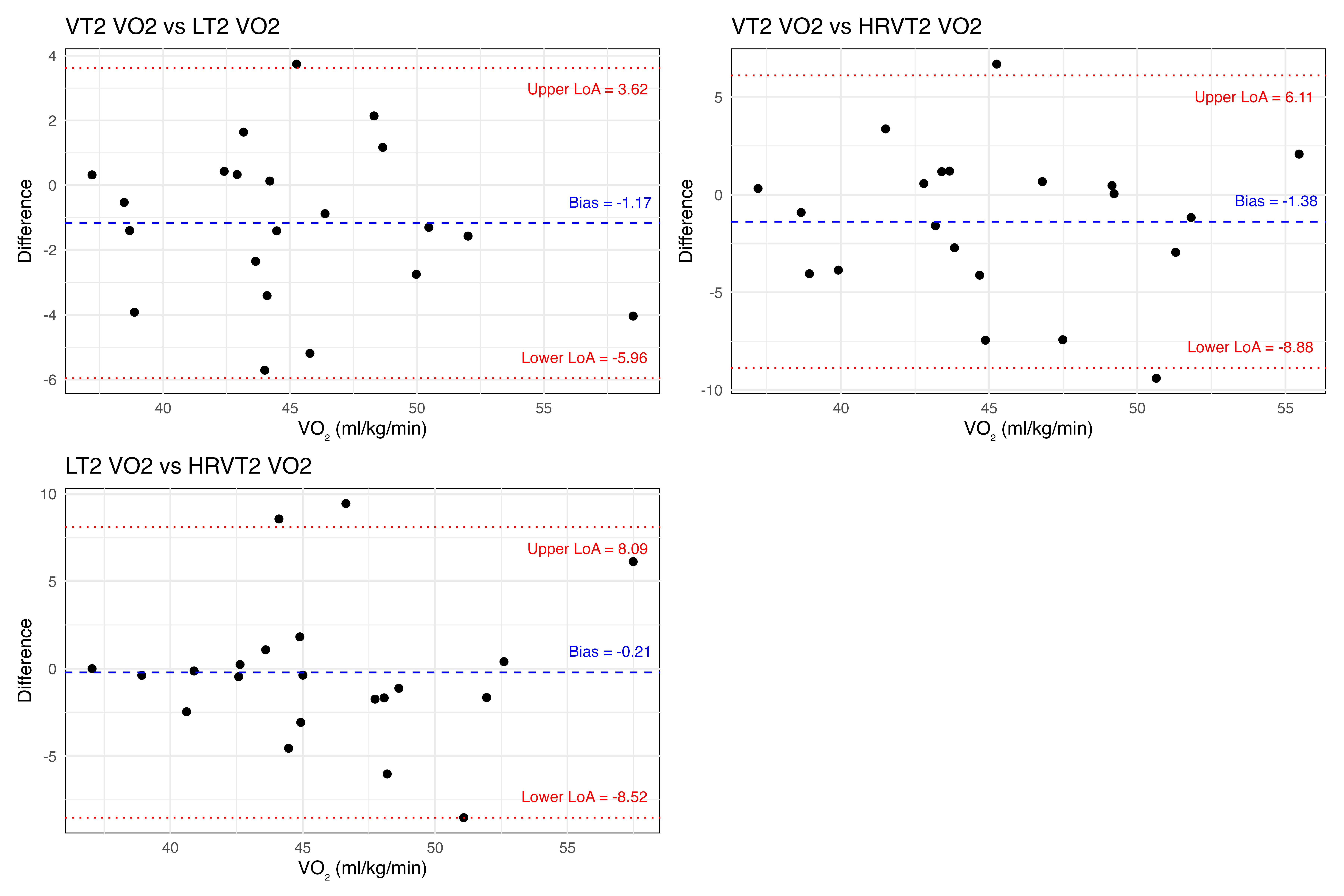

Supplement: Supplementary file 1 — Figure S1. (A) Bland–Altman plots of HRVT1(pers) versus VT1 versus LT1 for HR. HR, heart rate; HRVT1, first heart rate variability threshold; HRVT1pers, first personalized heart rate variability threshold; LOA, limits of agreement; LT1, first lactate threshold; VT1, first ventilatory threshold. (B) Bland–Altman plots of HRVT1(pers) versus VT1 versus LT1 for PO. HRVT1, first heart rate variability threshold; HRVT1pers, first personalized heart rate variability threshold; LOA, limits of agreement; LT1, first lactate threshold; PO, power output; VT1, first ventilatory threshold. (C) Bland–Altman plots of HRVT1(pers) versus VT1 versus LT1 for VO2. HRVT1, first heart rate variability threshold; HRVT1pers, first personalized heart rate variability threshold; LOA, limits of agreement; LT1, first lactate threshold; VO2, oxygen uptake; VT1, first ventilatory threshold. Figure S2. (A) Bland–Altman plots of HRVT2 versus VT2 versus LT2 for HR. HR, heart rate; HRVT2, second heart rate variability threshold; LOA, limits of agreement; LT2, second lactate threshold; VT2, second ventilatory threshold. (B) Bland–Altman plots of HRVT2 versus VT2 versus LT2 for PO. HRVT2, second heart rate variability threshold; LOA, limits of agreement; LT2, second lactate threshold; PO, power output; VT2, second ventilatory threshold. (C) Bland–Altman plots of HRVT2 versus VT2 versus LT2 for VO2. HR, heart rate; HRVT2, second heart rate variability threshold; LOA, limits of agreement; LT2, second lactate threshold; PO, power output; VO2, oxygen uptake; VT2, second ventilatory threshold. [file PHY2-14-e70777-s001.zip › PHYSREP-2025-12-1086-s06.png]

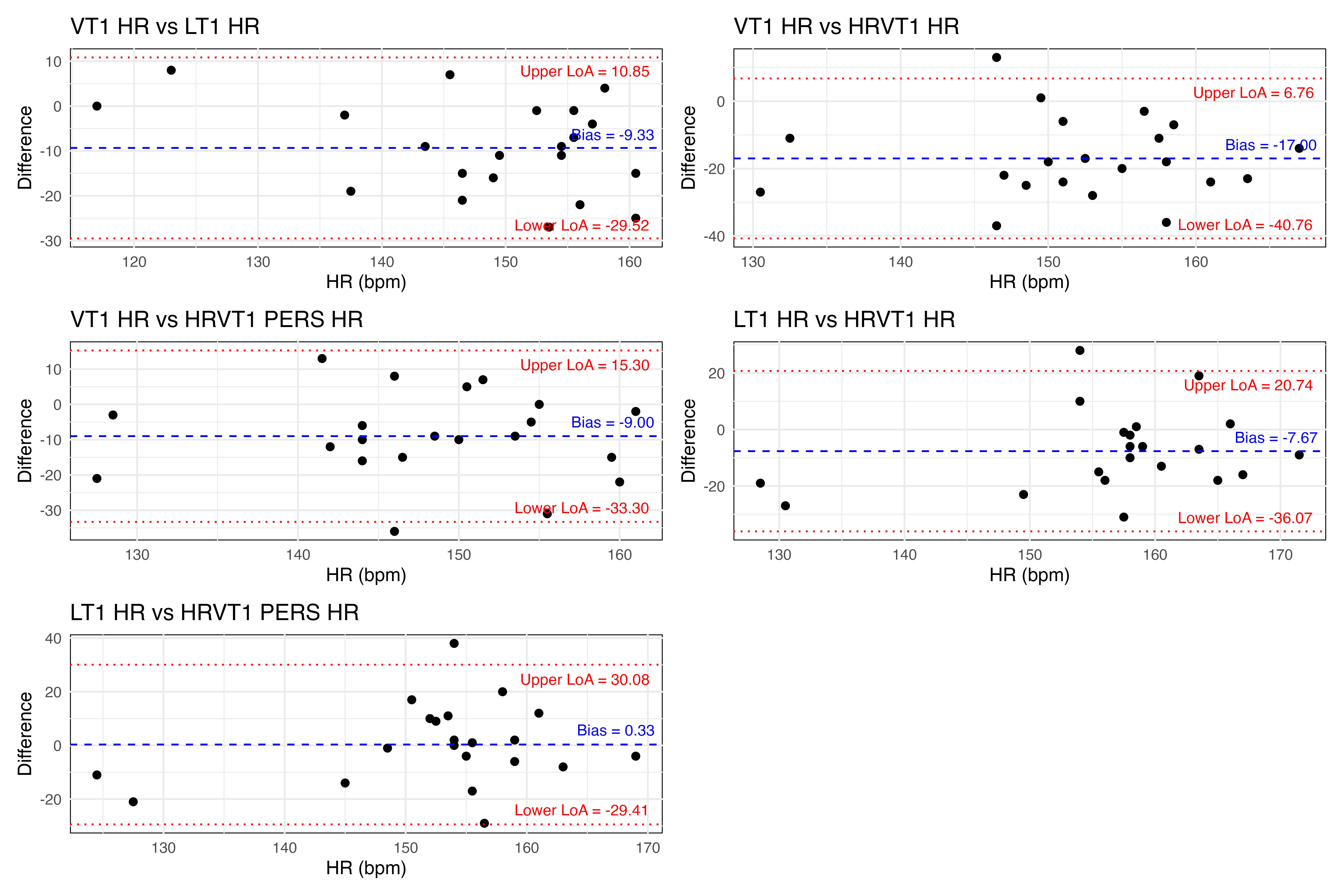

Supplement: Supplementary file 1 — Figure S1. (A) Bland–Altman plots of HRVT1(pers) versus VT1 versus LT1 for HR. HR, heart rate; HRVT1, first heart rate variability threshold; HRVT1pers, first personalized heart rate variability threshold; LOA, limits of agreement; LT1, first lactate threshold; VT1, first ventilatory threshold. (B) Bland–Altman plots of HRVT1(pers) versus VT1 versus LT1 for PO. HRVT1, first heart rate variability threshold; HRVT1pers, first personalized heart rate variability threshold; LOA, limits of agreement; LT1, first lactate threshold; PO, power output; VT1, first ventilatory threshold. (C) Bland–Altman plots of HRVT1(pers) versus VT1 versus LT1 for VO2. HRVT1, first heart rate variability threshold; HRVT1pers, first personalized heart rate variability threshold; LOA, limits of agreement; LT1, first lactate threshold; VO2, oxygen uptake; VT1, first ventilatory threshold. Figure S2. (A) Bland–Altman plots of HRVT2 versus VT2 versus LT2 for HR. HR, heart rate; HRVT2, second heart rate variability threshold; LOA, limits of agreement; LT2, second lactate threshold; VT2, second ventilatory threshold. (B) Bland–Altman plots of HRVT2 versus VT2 versus LT2 for PO. HRVT2, second heart rate variability threshold; LOA, limits of agreement; LT2, second lactate threshold; PO, power output; VT2, second ventilatory threshold. (C) Bland–Altman plots of HRVT2 versus VT2 versus LT2 for VO2. HR, heart rate; HRVT2, second heart rate variability threshold; LOA, limits of agreement; LT2, second lactate threshold; PO, power output; VO2, oxygen uptake; VT2, second ventilatory threshold. [file PHY2-14-e70777-s001.zip › PHYSREP-2025-12-1086-s01.png]

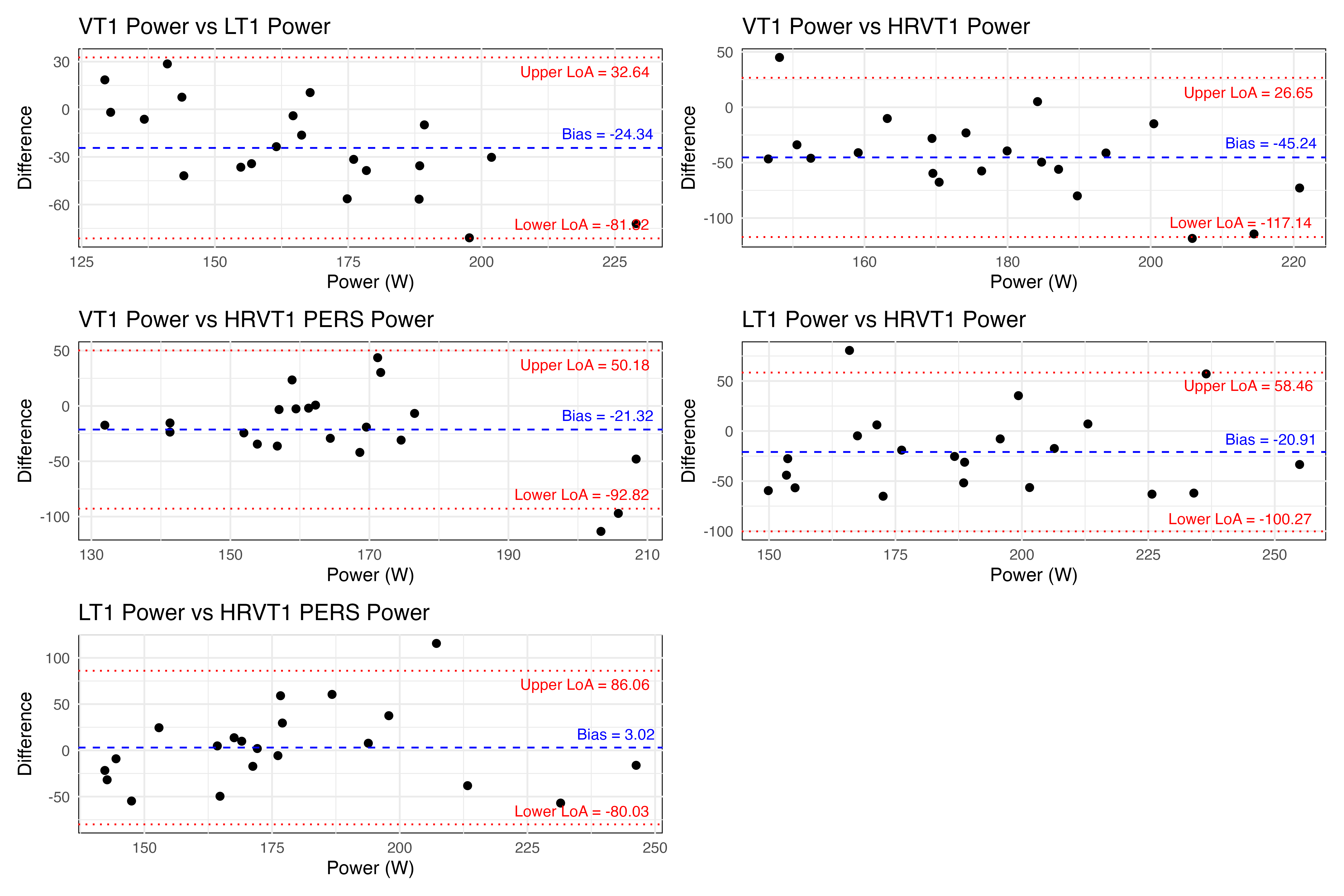

Supplement: Supplementary file 1 — Figure S1. (A) Bland–Altman plots of HRVT1(pers) versus VT1 versus LT1 for HR. HR, heart rate; HRVT1, first heart rate variability threshold; HRVT1pers, first personalized heart rate variability threshold; LOA, limits of agreement; LT1, first lactate threshold; VT1, first ventilatory threshold. (B) Bland–Altman plots of HRVT1(pers) versus VT1 versus LT1 for PO. HRVT1, first heart rate variability threshold; HRVT1pers, first personalized heart rate variability threshold; LOA, limits of agreement; LT1, first lactate threshold; PO, power output; VT1, first ventilatory threshold. (C) Bland–Altman plots of HRVT1(pers) versus VT1 versus LT1 for VO2. HRVT1, first heart rate variability threshold; HRVT1pers, first personalized heart rate variability threshold; LOA, limits of agreement; LT1, first lactate threshold; VO2, oxygen uptake; VT1, first ventilatory threshold. Figure S2. (A) Bland–Altman plots of HRVT2 versus VT2 versus LT2 for HR. HR, heart rate; HRVT2, second heart rate variability threshold; LOA, limits of agreement; LT2, second lactate threshold; VT2, second ventilatory threshold. (B) Bland–Altman plots of HRVT2 versus VT2 versus LT2 for PO. HRVT2, second heart rate variability threshold; LOA, limits of agreement; LT2, second lactate threshold; PO, power output; VT2, second ventilatory threshold. (C) Bland–Altman plots of HRVT2 versus VT2 versus LT2 for VO2. HR, heart rate; HRVT2, second heart rate variability threshold; LOA, limits of agreement; LT2, second lactate threshold; PO, power output; VO2, oxygen uptake; VT2, second ventilatory threshold. [file PHY2-14-e70777-s001.zip › PHYSREP-2025-12-1086-s02.png]
